# Cluster analysis of splenocyte microRNAs in the pig reveals key signal regulators of immunomodulation in the host during acute and chronic *Toxoplasma gondii* infection

**DOI:** 10.1186/s13071-022-05164-3

**Published:** 2022-02-17

**Authors:** Zhaofeng Hou, Hui Zhang, Kangzhi Xu, Shifan Zhu, Lele Wang, Dingzeyang Su, Jiantao Liu, Shijie Su, Dandan Liu, Siyang Huang, Jinjun Xu, Zhiming Pan, Jianping Tao

**Affiliations:** 1 College of Veterinary Medicine, Yangzhou University, Yangzhou, 225009 People’s Republic of China; 2 Jiangsu Co-Innovation Center for Prevention and Control of Important Animal Infectious Diseases and Zoonosis, Yangzhou, 225009 People’s Republic of China; 3 Jiangsu Key Laboratory of Zoonosis, Yangzhou, 225009 People’s Republic of China; 4 YEBIO Bioengineering Co., Ltd. of QINGDAO, Qingdao, 266109 People’s Republic of China

**Keywords:** *Toxoplasma gondii*, microRNA, Clustering analysis, Immunomodulation, Acute and chronic infection

## Abstract

**Background:**

*Toxoplasma gondii* is an obligate intracellular protozoan parasite that can cause a geographically widespread zoonosis. Our previous splenocyte microRNA profile analyses of pig infected with *T. gondii* revealed that the coordination of a large number of miRNAs regulates the host immune response during infection. However, the functions of other miRNAs involved in the immune regulation during *T. gondii* infection are not yet known.

**Methods:**

Clustering analysis was performed by *K*-means, self-organizing map (SOM), and hierarchical clustering to obtain miRNA groups with the similar expression patterns. Then, the target genes of the miRNA group in each subcluster were further analyzed for functional enrichment by Gene Ontology (GO), Kyoto Encyclopedia of Genes and Genomes (KEGG), and Reactome pathway to recognize the key signaling molecules and the regulatory signatures of the innate and adaptive immune responses of the host during *T. gondii* infection.

**Results:**

A total of 252 miRNAs were successfully divided into 22 subclusters by *K*-means clustering (designated as K1–K22), 29 subclusters by SOM clustering (designated as SOM1–SOM29), and six subclusters by hierarchical clustering (designated as H1–H6) based on their dynamic expression levels in the different infection stages. A total of 634, 660, and 477 GO terms, 15, 26, and 14 KEGG pathways, and 16, 15, and 7 Reactome pathways were significantly enriched by *K*-means, SOM, and hierarchical clustering, respectively. Of note, up to 22 miRNAs mainly showing downregulated expression at 50 days post-infection (dpi) were grouped into one subcluster (namely subcluster H3-K17-SOM1) through the three algorithms. Functional analysis revealed that a large group of immunomodulatory signaling molecules were controlled by the different miRNA groups to regulate multiple immune processes, for instance, IL-1-mediated cellular response and Th1/Th2 cell differentiation partly depending on Notch signaling transduction for subclusters K1 and K2, innate immune response involved in neutrophil degranulation and TLR4 cascade signaling for subcluster K15, B cell activation for subclusters SOM17, SOM1, and SOM25, leukocyte migration, and chemokine activity for subcluster SOM9, cytokine–cytokine receptor interaction for subcluster H2, and interleukin production, chemotaxis of immune cells, chemokine signaling pathway, and C-type lectin receptor signaling pathway for subcluster H3-K17-SOM1.

**Conclusions:**

Cluster analysis of splenocyte microRNAs in the pig revealed key regulatory properties of subcluster miRNA molecules and important features in the immune regulation induced by acute and chronic *T. gondii* infection. These results contribute new insight into the identification of physiological immune responses and maintenance of tolerance in pig spleen tissues during *T. gondii* infection.

**Graphical Abstract:**

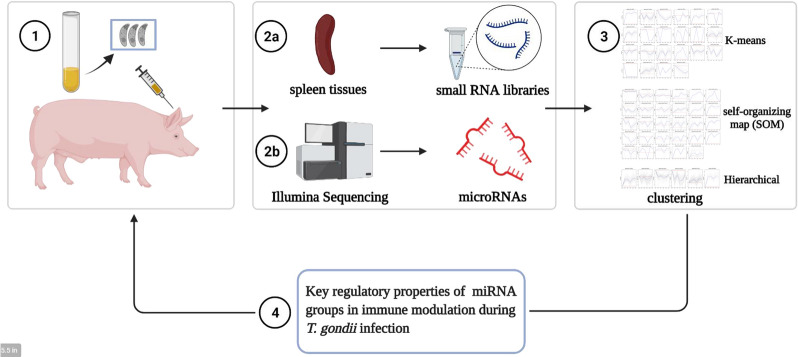

**Supplementary Information:**

The online version contains supplementary material available at 10.1186/s13071-022-05164-3.

## Background

*Toxoplasma gondii* (*T. gondii*) is an obligate intracellular protozoan parasite that can cause geographically widespread zoonosis. Humans and virtually all warm-blooded species can be intermediate hosts and become infected by ingestion of food and water contaminated with the sporulated *T. gondii* oocysts by consumption of tissue cysts in infected animal tissues, or congenitally. Ingestion of porcine meat containing persistent tissue cysts is considered to be a major source of *T. gondii* infection in humans [1]. Besides the adverse clinical consequences for humans, *T. gondii* can cause disseminated and fatal infection in pigs. In fact, this parasite is known to cause different types of infection clinically in pigs, including acute onset of toxoplasmosis or asymptomatic infection, which are related to the amount, virulence, and genotypes of *T. gondii*. *Toxoplasma gondii* is genetically and biologically divergent. There are different genotypes of *T. gondii* from pigs in different regions. The Chinese I genotype strain (ToxoDB #9) is predominant in China [2]. Previous studies have demonstrated that virulence phenotypes in mice differed among strains sharing this genotype [3, 4]. The unique molecular basis of the genotype may lead to many differences in host resistance characteristics.

MicroRNAs (miRNA) are a class of endogenous non-protein-coding RNAs that can control gene expression at the post-transcriptional level through mRNA cleavage, mRNA degradation, or translational repression [5]. Mature miRNAs can directly affect the expression of thousands of genes and primarily serve as negative regulators of the host response during parasite infection [6]. During invasion of the host cells, a set of specialized secretory organelles of *T. gondii* are used to interfere with the host cell signaling pathways and alter the host defense systems by hijacking host miRNA pathways [7, 8]. The miRNAs showing differential expression between infected and uninfected cells modulated crucial host cellular targets that participate in determining the success of *T. gondii* infection [9], and subverted the host innate and adaptive immunity, underscoring the importance of miRNAs in parasite–host interactions [10, 11].

Increasing functional reports have demonstrated the key role of miRNAs in regulating host immunity induced by *T. gondii*. This parasite can suppress nuclear factor kappa B (NF-κB) activation by inducing the expression of miR-146a in the host [12]. The absence of miR-146a expression affects parasitic load, leading to significant differences in interferon gamma (IFN-γ) production and long-term survival of infected mice [13]. miR-155 is associated with recruiting Treg and CD8^+^ cells in *T. gondii* infection [11]. As an early response to infection, delayed IL-12 production in mouse macrophages was shown to be regulated by miR-187 to evade host immune surveillance [14]. Nevertheless, our and other studies have found that *T. gondii* regulates the host immune response depending on the coordination of a large number of miRNAs [6, 9, 15–17], and it is difficult for a single or a few miRNAs to regulate and determine the nature of the immune response during infection. Although a considerable number of miRNAs have been discovered, and a significant fraction of the miRNA repertoire of any species is conserved with other species [18], there are still miRNAs from different species that have not been identified or discovered, or where the known miRNA functions have not been fully explored. Highly similar expression may suggest similar function, and cluster analysis of miRNA populations based on the expression levels in different experimental backgrounds may help to distinguish novel miRNA functions or unknown functions of known miRNAs [19, 20].

In order to further investigate the unknown functions of miRNAs of the pig following *T. gondii* infection, we identified miRNAs dynamically expressed in pig spleen tissues during the different infection stages. Cluster analysis was then performed by *K*-means, self-organizing map (SOM), and hierarchical clustering to obtain miRNA groups with similar expression patterns. The target genes of the miRNA group in each subcluster were further analyzed for functional enrichment by Gene Ontology (GO), the Kyoto Encyclopedia of Genes and Genomes (KEGG), and the Reactome pathway to recognize the key signaling molecules and the regulatory signatures of the innate and adaptive immune responses of the host during *T. gondii* infection, which would assist in planning preventive measures against *T. gondii* infection in pigs.

## Methods

### Parasites and animals

The *T. gondii* YZ-1 strain that was used to infect pigs in this study was identified as genotype ToxoDB #9 (Chinese I) and shown to be virulent in mice in our previous study [4]. Twenty-four 5-week-old commercial specific-pathogen-free swine were obtained from the Jiangsu Meilin Animal Husbandry Co., Ltd. (Jiangsu, China). Parasite challenge experiments were performed according to our previous description [15].

### Small RNA sequencing and bioinformatics analysis

The samples and their small RNA sequencing data used in the current study were derived from our previous study [15]. Briefly, spleen samples were collected from three pigs from the infection and control groups at 10, 25, and 50 days post-infection (dpi). Six small RNA libraries including SI-10, SI-25, and SI-50 in the spleen-infected (SI) group and SC-10, SC-25, and SC-50 in the spleen control (SC) group were used for further analysis to investigate the biological roles of miRNAs for the current study. Full details of the sequence data have been submitted to the Gene Expression Omnibus public database (GEO; http://www.ncbi.nlm.nih.gov/geo/) with GEO accession number GSE113130, and the raw data are available in the NCBI Sequence Read Archive under the accession number SRP139950.

### Clustering of miRNAs

TPM (transcript per million clean tags) was used to assess the expression levels of miRNAs, and principal component analysis (PCA) was performed in the R platform (http://www.ehbio.com/ImageGP/index.php/Home/Index/) using a log_2_ transformation of TPM-normalized data. Differential expression between groups was analyzed using the DEGseq (2010) R package. *P*-values were corrected to obtain the *q*-value using the Benjamini–Hochberg method, and a *q*-value < 0.01 and |log_2_(fold change)| > 1 was set as the threshold for significantly differential expression by default.

To compare expression patterns of individual miRNAs and to determine the clustering mode of miRNAs during the different infection stages, miRNA clustering was performed through calculation by log_10_(TPM + 1) based on the expression levels of miRNAs in the six libraries. The targeted data used for clustering was the union of differential miRNAs, and log_2_(ratios) was defined as the relative expression level of miRNA, which was used to analyze clustering of miRNAs. The distance between each miRNA was calculated using the corresponding distance algorithm, and then the relative distance between miRNAs was calculated through repeated iterations. The different subclusters were finally obtained according to the relative distance of miRNAs. Euclidian distance was used to calculate the distance within and among clusters. In this study, clustering of the miRNAs between different infection stages was analyzed using *K*-means, SOM, and hierarchical clustering from base package stats of R.

### Functional enrichment analysis

The target genes of the miRNAs in different cluster groups were predicted using miRanda and RNAhybrid. GO, KEGG, and Reactome pathway [21] enrichment analyses were performed to identify the cellular function and molecular pathways regulated by the miRNAs. In addition, some miRNA-regulated pathways were analyzed by the KEGG map annotations [22]. To gain insight into the interactions between miRNAs and target genes related to immune regulation induced by *T. gondii* infection, miRNA-gene networks were created and visualized using Cytoscape 3.4.0 software [23].

## Results

**Fig. 1:**
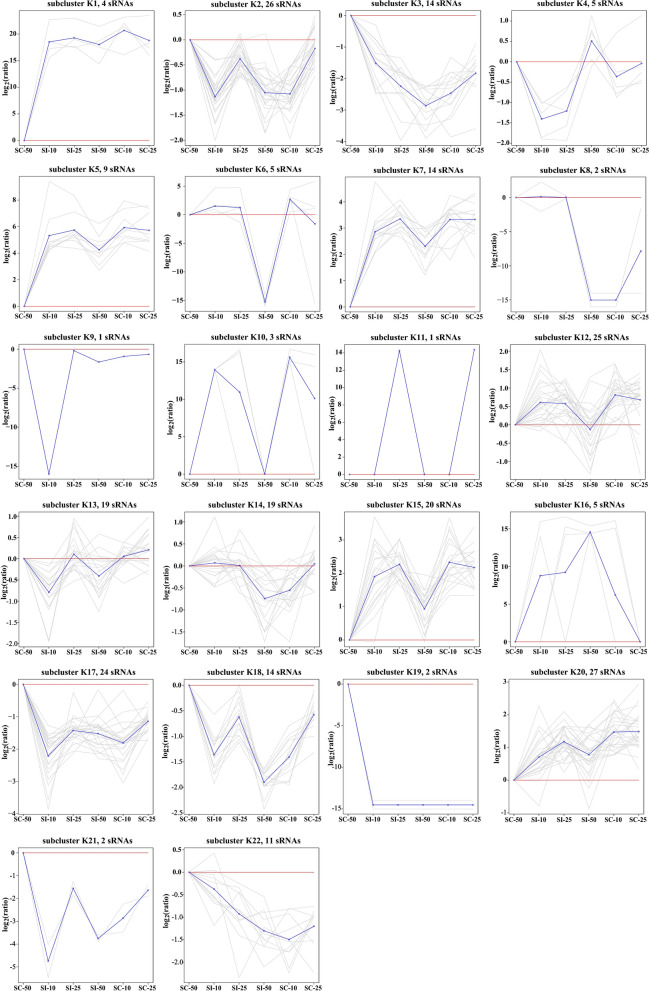
*K*-means clustering patterns of pig splenocyte miRNAs. All miRNAs were clustered into 22 groups using *K*-means clustering and visualized with TM4 software. The blue line shows average expression *z*-scores to visualize the dominant expression trend of each subcluster. Each line in the figure represents an expression value of the corresponding miRNA

**Fig. 2:**
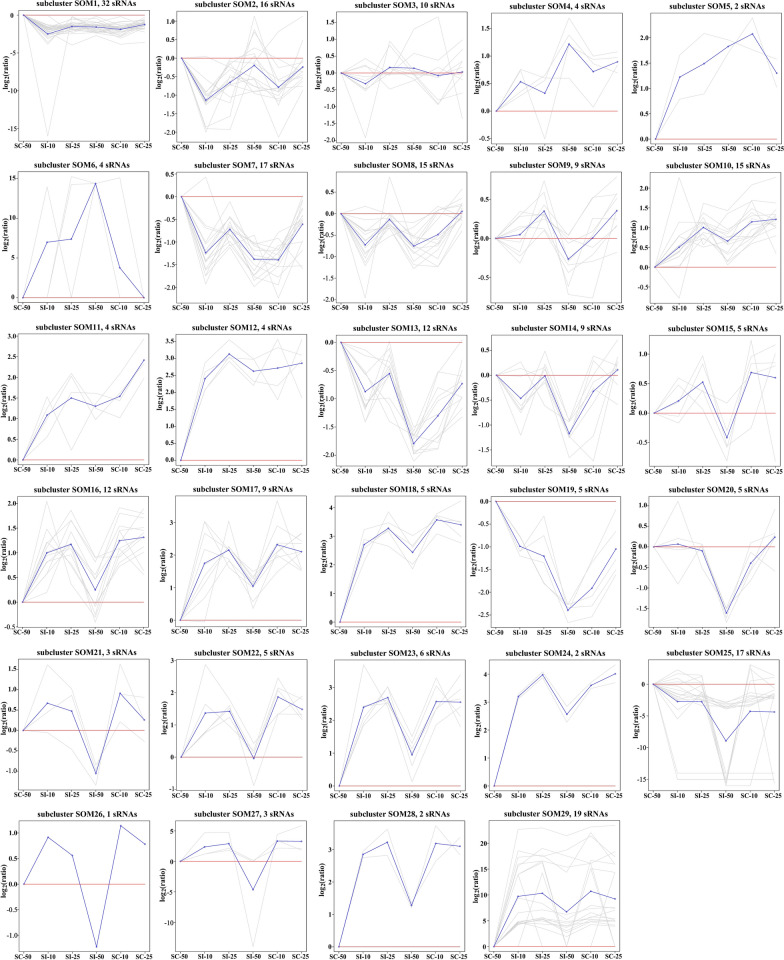
SOM clustering patterns of pig splenocyte miRNAs. All miRNAs were clustered into 29 groups using SOM clustering and visualized with TM4 software. The blue line shows average expression *z*-scores to visualize the dominant expression trend of each subcluster. Each line in the figure represents an expression value of the corresponding miRNA

**Fig. 3:**
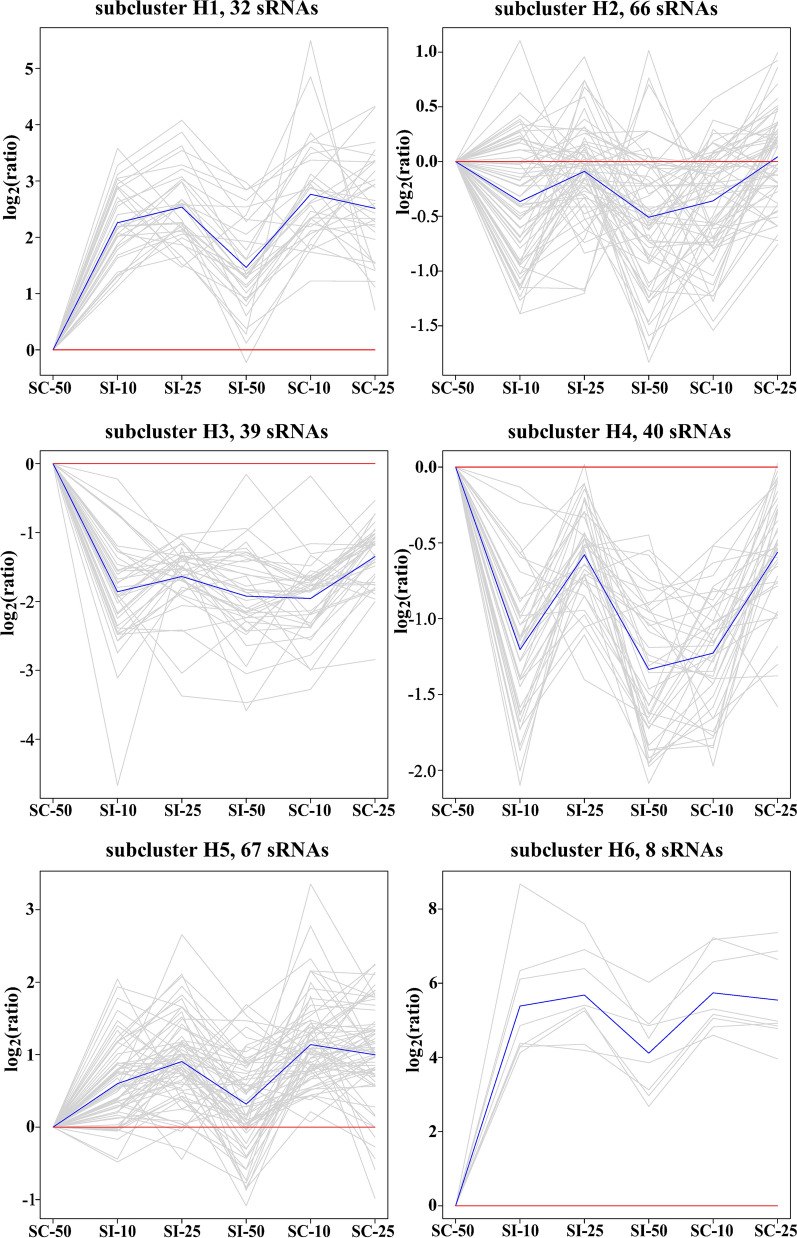
Hierarchical clustering patterns of pig splenocyte miRNAs. All miRNAs were clustered into six groups using hierarchical clustering and visualized with TM4 software. The blue line shows average expression *z*-scores to visualize the dominant expression trend of each subcluster. Each line in the figure represents an expression value of the corresponding miRNA

A total of 285 unique pig-encoded miRNAs, including 260 known and 25 novel mature miRNAs, were found to be shared amongst the six libraries. A PCA plot of individual small RNA libraries is shown in Additional file 1: Fig. S1, which indicates that the infected samples can be clustered. Of these mature miRNAs, 252 miRNAs (235 known and 17 novel miRNAs) were successfully divided into 22 subclusters by *K*-means clustering (designated as K1–K22), 29 subclusters by SOM clustering (designated as SOM1–SOM29), and six subclusters by hierarchical clustering (designated as H1–H6), each characterized by a unique expression pattern. The patterns corresponding to individual clusters are visualized as line plots (Figs. 1, 2, and 3). A total of 106 known and three novel miRNAs were significantly differentially expressed between the infected and control samples at the three time points (*q* < 0.001). A total of 4908 predicted target genes of 224 miRNAs were in agreement with the overlap of two prediction methods (miRanda and RNAhybrid), and these constituted our conservative set. However, 28 miRNAs had no target genes in agreement with the overlap of these two methods.

### *K*-means clustering

For *K*-means clustering, the number of miRNAs and their expression trends at different infection times in different subclusters are shown in Fig. 1. Subclusters K1–K22 had different numbers of subjects, and subclusters K9 and K11 had only one subject, with miR-216 and miR-105-1, respectively, while subcluster K20 had the greatest number of subjects, with 27 miRNAs, followed by subcluster K2 that had 26 miRNAs. Additionally, 109 identified differentially expressed miRNAs (DEMs) were found to be distributed in 15 subclusters, namely K1–5, K7, K12–15, K17, K18, and K20–22. Seven subclusters (K6, K8–11, K16, and K19) did not exhibit the differential miRNA expression patterns specific to any infection stage.

In GO enrichment, 513 biological processes (BP), 58 cellular components (CC), and 63 molecular functions (MF) were significantly enriched GO terms (*q* < 0.05) by the target genes of the miRNAs in nine K subclusters, including K1–3, K12–15, K17, and K18. Eight BP terms, including regulation of cellular process (GO: 0050794), cell communication (GO: 0007154), biological regulation (GO: 0065007), metabolic process (GO: 0008152), signaling (GO: 0023052), regulation of BP (GO: 0050789), response to stimulus (GO: 0050896), and cellular process (GO: 0009987), were shared amongst these nine K subclusters. Subcluster K13, containing 19 miRNAs, was found to enrich into the least number of GO terms, with 32 terms, including 27 BP, two CC, and three MF (Table 1). Subcluster K15, containing 20 miRNAs, was found to enrich into the greatest number of GO terms, with 443 terms, including 361 BP, 38 CC, and 44 MF (Table 1). The enriched GO terms were related to signal transduction, response to stimulus/stress, immune process, apoptotic process, metabolic process, and biosynthetic process for BP, and catalytic activity, GTPase activity, and nucleotide binding for MF (Additional file 2: Table S1). The GO terms related to defense response, innate immune response, NF-κB transcription, tumor necrosis factor (TNF)-mediated signaling pathway, and inflammatory response involving immune response-regulating cell surface receptor signaling pathway, cytokine secretion and leukocyte activation, aggregation and migration were only enriched by the K15 subcluster. Additionally, GO terms related to angiogenesis, interleukin-1 (IL-1)-mediated signaling pathway, Notch signaling pathway, and vasculature development were found to be subcluster K1-specific (Additional file 2: Table S1). Amino acid transport-related terms were the most frequently represented objects in subcluster K2-specific GO terms, cell cycle process and DNA replication-related terms for K12, and protein localization and transport for K18 (Additional file 2: Table S1).

**Table 1:** The number of the significantly enriched functional terms for predicted target genes of miRNAs in different subclusters by *K*-means clustering

Enrichment information	Subclusters									Total
	K1	K2	K3	K12	K13	K14	K15	K17	K18	
miRNA	4	26	14	25	19	19	20	24	14	165
Target genes	103	1223	947	1577	884	1236	1292	1005	950	4364
GO										
Biological process	188	192	69	169	27	32	361	62	42	513
Cellular component	13	25	17	21	2	20	38	10	9	58
Molecular function	11	19	8	16	3	10	44	8	3	63
Total	212	236	94	206	32	62	443	80	54	634
KEGG	4	6	0	2	0	1	3	3	0	15
Reactome	5	4	1	1	0	0	7	1	0	16

In the KEGG enrichment, 15 KEGG pathways involving cellular processes (peroxisome, regulation of actin cytoskeleton, focal adhesion, phagosome), human diseases [dilated cardiomyopathy (DCM), toxoplasmosis, hepatitis B, Chagas disease (American trypanosomiasis), pertussis, proteoglycans in cancer], signal transduction (MAPK signaling pathway, TNF signaling pathway), immune system (Th1 and Th2 cell differentiation, hematopoietic cell lineage), and metabolism (fatty acid degradation) were significantly enriched by six subclusters (*q* < 0.05), including K1, K2, K12, K14, K15, and K17 (Table 1, Additional file 3: Table S2). Of these, peroxisome (ssc04146) was shared amongst K subclusters K1, K15, and K17. Subcluster K2, containing 26 miRNAs, was found to enrich into the greatest number (6) of KEGG pathways. Of these, Th1 and Th2 cell differentiation (ssc04658) and toxoplasmosis (ssc05145) were subcluster K2-specific (Additional file 3: Table S2).

In the Reactome pathway enrichment, 16 Reactome pathways involving metabolism, immune system, cell–cell communication, and signal transduction were significantly enriched (*q* < 0.05) by subclusters K1, K2, K3, K12, K15, and K17 (Table 1, Additional file 4: Table S3). Interestingly, all of the metabolism-related pathways, including biosynthesis of phosphatidylcholine and glycerophospholipid (R-SSC-1483191, R-SSC-1483206) and metabolism of phospholipid and cobalamin (R-SSC-1483257, R-SSC-196741) were found only in subcluster K1. All of the cell connection-related pathways, including cell junction organization (R-SSC-446728), cell–cell communication (R-SSC-1500931), and cell–extracellular matrix interactions (R-SSC-446353), were found only in subcluster K2. The immune system-related pathways were found to be the most frequently represented pathways, which were found to distribute in subclusters K2, K3, K12, and K15 (Additional file 4: Table S3). Additionally, no significantly enriched GO, KEGG, or Reactome terms were found in subclusters K4–11, K16, and K19–22.

### SOM clustering

For SOM clustering, the number of miRNAs and their expression trends at different infection times in different subclusters are shown in Fig. 2. Subclusters SOM1–SOM29 had different numbers of subjects, and subcluster SOM26 had only one subject, with miR-326, while subcluster SOM1 had the greatest number of subjects, with 32 miRNAs, followed by subcluster SOM29 that had 19 miRNAs. Additionally, 109 identified DEMs were found to be distributed in 26 subclusters, namely SOM1–5, SOM7–14, SOM16–25, and SOM27–29. Three subclusters (SOM6, SOM15, and SOM26) did not exhibit the differential miRNA expression patterns specific to any infection stage.

In GO enrichment, a total of 660 GO terms, including 552 BP, 46 CC and 62 MF, were significantly enriched (*q* < 0.05) by the target genes of the miRNAs from 11 SOM subclusters, including SOM1, SOM8–10, SOM12–14, SOM17, SOM18, SOM25, and SOM29 (Table 2). Response to stimulus (GO: 0050896) was shared amongst nine SOM subclusters (SOM1, SOM8–10, SOM13, SOM14, SOM17, SOM25, and SOM29) and was found to be the most frequently represented term. Subcluster SOM25, containing 17 miRNAs, was found to enrich into the most number of GO terms, with 457 terms, including 386 BP, 29 CC and 42 MF (Table 2), of which 110 terms were only found in this subcluster and were mostly associated with calcium ion transport, stress-activated responses, MAPK cascade signaling, and cell apoptosis (Additional file 5: Table S4). Interestingly, the specific GO terms were found to be enriched by some subclusters. Ion binding- and ion transport-related terms were only found in subclusters SOM8 and SOM12, respectively. All four terms that were subcluster SOM9-specific were related to leukocyte migration and chemotaxis, including lymphocyte migration (GO: 0072676), leukocyte migration (GO: 0050900), chemokine activity (GO: 0008009), and chemokine receptor binding (GO: 0042379). Furthermore, GO terms related to cytokine production and secretion, IL-1 production, GTPase activity, and NF-κB signaling were found to be the most frequently represented objects in the subcluster SOM17-specific terms (Additional file 5: Table S4).

**Table 2:** The significantly enriched functional terms for predicted target genes of miRNAs in different subclusters by SOM clustering

Enrichment information	Subclusters											Total
	SOM1	SOM8	SOM9	SOM10	SOM12	SOM13	SOM14	SOM17	SOM18	SOM25	SOM29	
miRNA	32	15	9	15	4	12	9	9	5	17	19	146
Target gene	1227	1266	245	485	85	734	719	1116	84	1745	498	4189
GO												
Biological process	108	43	15	2	6	47	64	342	9	386	188	552
Cellular component	15	18	0	1	1	7	9	34	0	29	4	46
Molecular function	13	10	2	0	4	2	2	38	0	42	25	62
Total	136	71	17	3	11	56	75	414	9	457	217	660
KEGG	3	1	0	0	1	1	6	2	0	9	10	26
Reactome	0	1	1	0	0	0	0	4	0	9	4	15

In the KEGG enrichment, 26 KEGG pathways involving cellular processes, signal transduction, human diseases, and organismal systems were significantly enriched by eight subclusters (*q* < 0.05), including SOM1, SOM8, SOM12–14, SOM17, SOM25, and SOM29 (Table 2, Additional file 6: Table S5). Of these, 19 KEGG pathways were classified into human diseases (11) and signal transduction (8). The MAPK signaling pathway (ssc04010) and TNF signaling pathway (ssc04668) were shown to be the pathways most frequently enriched amongst SOM subclusters (Additional file 6: Table S5). The MAPK signaling pathway was shared amongst subclusters SOM8, SOM25, and SOM29, and the TNF signaling pathway was common to subclusters SOM14, SOM25, and SOM29. Notably, subcluster SOM29, containing 19 miRNAs, was found to enrich into the greatest number (10) of KEGG pathways, followed by SOM25 with enrichment into nine KEGG pathways. Of the 10 KEGG pathways for subcluster SOM29 and nine KEGG pathways for subcluster SOM25, eight and five were found to be subcluster SOM29- and SOM25-specific, respectively (Additional file 6: Table S5).

In the Reactome pathway enrichment, 15 Reactome pathways involving cell–cell communication, gene expression, immune system, signal transduction, developmental biology, DNA repair, and hemostasis were significantly enriched by subclusters SOM8, SOM9, SOM17, SOM25, and SOM29 (*q* < 0.05). SOM25 was found to enrich into the greatest number (nine) of Reactome pathways (Table 2). Notably, all of the gene expression- and DNA repair-related pathways, including RNA polymerase III transcription initiation from type 1 promoter (R-SSC-76061), RNA polymerase III transcription initiation from type 2 promoter (R-SSC-76066), RNA polymerase III transcription (R-SSC-74158), RNA polymerase III transcription initiation (R-SSC-76046), gap-filling DNA repair synthesis and ligation in TC-NER (R-SSC-6782210), and dual incision in TC-NER (R-SSC-6782135), were enriched only by subcluster SOM25. Cytokine signaling in the immune system (R-SSC-1280215) was the most frequently represented pathway, which was found to distribute in subclusters SOM17, SOM25, and SOM29 (Additional file 7: Table S6). Additionally, no significantly enriched GO, KEGG, or Reactome terms were found in subclusters SOM2–7, SOM11, SOM15, SOM16, SOM19–24, SOM26, or SOM27.

### Hierarchical clustering

For hierarchical clustering, the number of miRNAs and their expression trends at different infection times in different subclusters are shown in Fig. 3. Subclusters H1–H6 had different numbers of subjects; subcluster H5 had the greatest number of subjects, with 67 miRNAs, while subcluster H6 had the least number of subjects, with eight miRNAs.

In GO enrichment, a total of 477 GO terms, including 371 BP, 46 CC and 60 MF, were significantly enriched (*q* < 0.05) by the target genes of the miRNAs from five H subclusters, including H1–H5 (Table 3). Of these, 41 GO terms were shared amongst five H subclusters (H1–H5), most of which were associated with cell communication, cellular process, and response to stimulus. Subcluster H5, containing 67 miRNAs, was found to have the greatest number of GO terms, with 376 terms, including 304 BP, 32 CC and 40 MF (Table 3), of which 94 terms were only found in this subcluster and were mostly associated with apoptotic process, DNA binding transcription factor activity, and ion homeostasis and transport (Additional file 8: Table S7). Additionally, further analysis revealed that some GO terms were subcluster-specific. For example, RNA splicing-related terms were the most frequently represented objects in the subcluster H1-specific terms, and lymphocyte activation-related terms were common in the subcluster H3-specific terms (Additional file 8: Table S7).

**Table 3:** The significantly enriched functional terms for predicted target genes of miRNAs in different subclusters by hierarchical clustering

Enrichment information	Subclusters					Total
	H1	H2	H3	H4	H5	
miRNA	32	66	39	40	67	244
Target gene	708	2771	1723	1913	2999	4908
GO						
Biological process	85	243	156	63	304	371
Cellular component	13	39	23	11	32	46
Molecular function	12	36	27	14	40	60
Total	110	318	206	88	376	477
KEGG	1	4	6	0	3	14
Reactome	1	3	1	1	4	7

In the KEGG enrichment, 14 KEGG pathways involving signal transduction, human diseases, and immune system were significantly enriched by four subclusters (*q* < 0.05), including H1–H3 and H5 (Table 3, Additional file 9: Table S8). Of these, seven KEGG pathways were classified into human diseases involving cancer (three), cardiovascular disease (one), immune disease (one), and infectious disease (two) (Additional file 9: Table S8). Subcluster H3, containing 39 miRNAs, was found to have the greatest number of KEGG pathways (six), followed by H2 with four KEGG pathways (Table 3).

In the Reactome pathway enrichment, seven Reactome pathways involving the immune system and autophagy were significantly enriched (*q* < 0.05) by subclusters H1–H5 (Table 3, Additional file 10: Table S9). Of these, the immune system (R-SSC-168256) pathway was shared amongst subclusters H2–H5. H5 was found to enrich into the greatest number (four) of Reactome pathways. It is worth mentioning that macroautophagy (R-SSC-1632852) was enriched only by subcluster H1. And innate immune system-related pathways, including innate immune system (R-SSC-168249), antimicrobial peptides (R-SSC-6803157), and toll-like receptor 4 (TLR4) cascade (R-SSC-166016), were enriched by the subclusters H2 and H5. Furthermore, two cytokine signaling in immune system-related pathways, including cytokine signaling in immune system (R-SSC-1280215) and signaling by ILs (R-SSC-449147), were found only in subcluster H5 (Additional file 10: Table S9). Additionally, no significantly enriched GO, KEGG, or Reactome terms were found in subcluster H6.

### The overlap of miRNAs among *K*-means clustering, SOM clustering, and hierarchical clustering

Considerable bias was found among *K*-means, SOM, and hierarchical clustering in regard to the representation of clustered genes for the three functional enrichments, mainly due to the differences in calculation methods for miRNA expression patterns. The clusters obtained by the three algorithms were compared to identify similar expression patterns; up to 22 miRNAs mainly showing downregulated expression at 50 dpi were identified into one subcluster (namely subcluster H3-K17-SOM1) through the three algorithms in this study, and were included in the subcluster K17 for *K*-means clustering, subcluster SOM1 for SOM clustering, and subcluster H3 for hierarchical clustering. In addition to novel-201, the remaining 21 miRNAs were classified into 12 miRNA families (let-7, miR-1, miR-10, miR-128, miR-129, miR-139, miR-1468, miR-27, miR-28, miR-30, miR-328, miR-455), of which six miRNA members, including let-7a, let-7c, let-7e, let-7f, let-7g, and let-7i, belonged to the let-7 family, and four miRNAs, including miR-30a-3p, miR-30b-3p, miR-30c-3p, and miR-30e-3p, belonged to the mir-30 family. A total of 860 target genes corresponding to 20 miRNAs (no containing novel-201 and miR-99a) in subcluster H3-K17-SOM1 were further used to analyze their cellular functions.

**Fig. 4:**
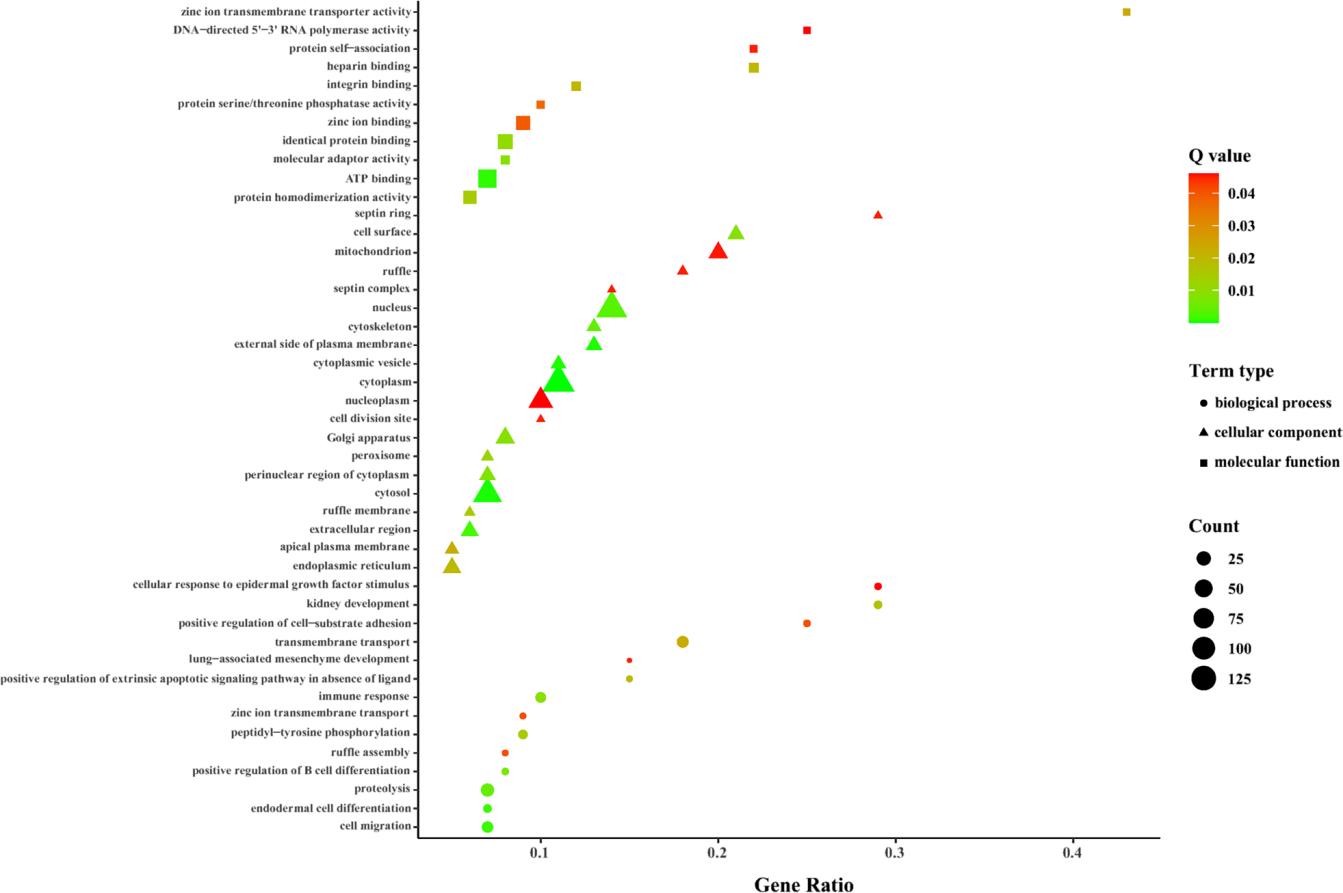
GO enrichment analysis for target genes of miRNAs in subcluster H3-K17-SOM1. The main GO terms of miRNAs in subcluster H3-K17-SOM1 are shown. The *x*-axis represents the gene ratio that indicates the ratio of target genes of miRNAs enriched in the terms among genes annotated in the terms. The sizes of the nodes represent term types

GO enrichment analysis revealed that a total of 400 GO terms were significantly enriched (*p* < 0.05) by the subcluster H3-K17-SOM1, and the main enriched GO terms are shown in Fig. 4. The target genes of miRNAs in this subcluster were involved in the apoptotic process, cell adhesion, GTPase activity, protein deubiquitination, and especially immune response including B cell differentiation, immunoglobulin production, inflammatory response, innate immune response, IL production, natural killer (NK) cell-mediated cytotoxicity, neutrophil chemotaxis, T cell cytokine production, T cell differentiation, and T cell proliferation (Additional file 11: Table S10). In addition, enrichment results showed that most of the GO terms (14 terms) of cellular components were related to mitochondrion, suggesting that many target genes of miRNAs in subcluster H3-K17-SOM1 located in mitochondrion may play important roles in mitochondrial function during *T. gondii* infection.

**Fig. 5:**
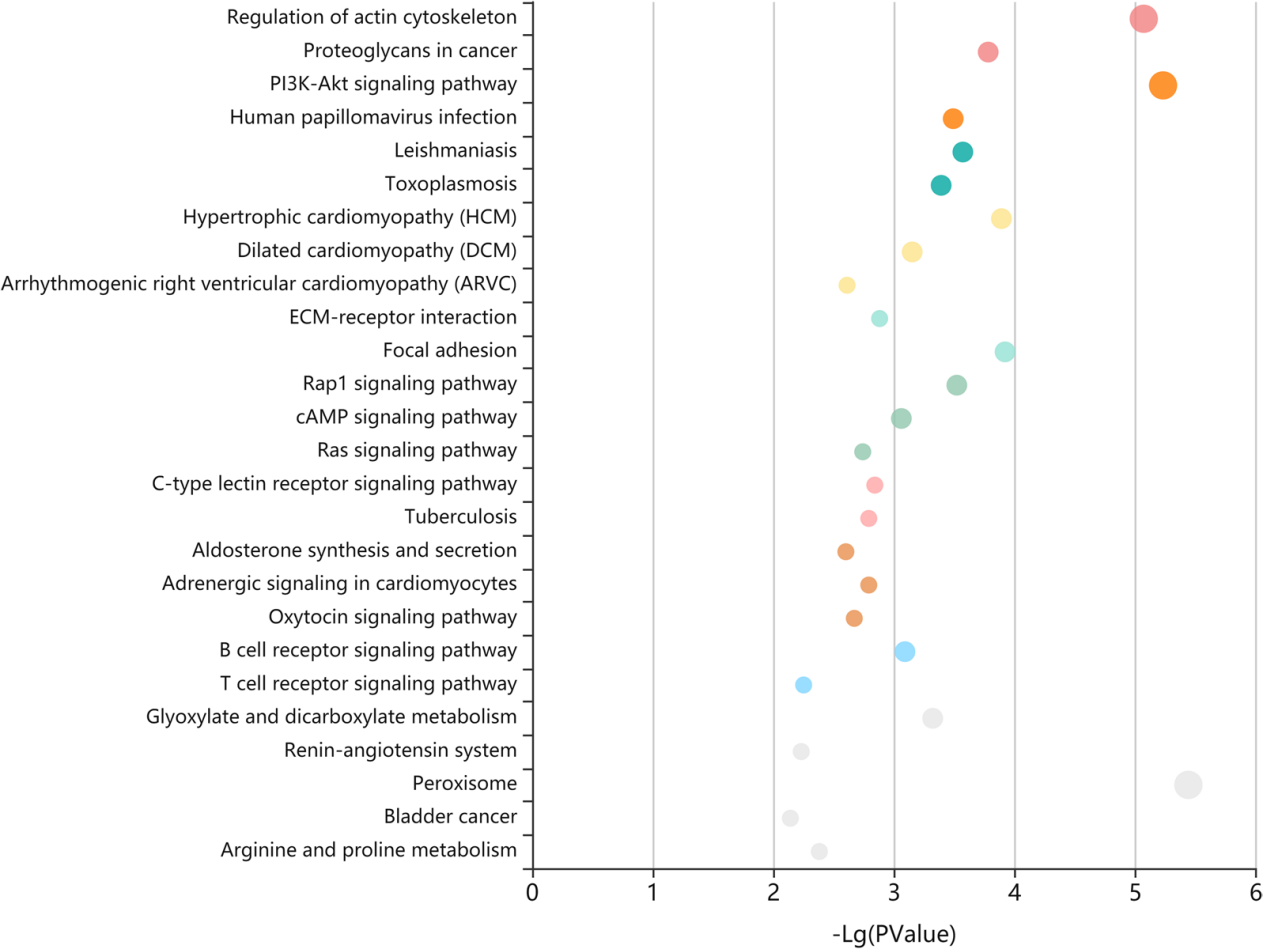
KEGG enrichment analysis for target genes of miRNAs in subcluster H3-K17-SOM1. The main KEGG pathways of miRNAs in subcluster H3-K17-SOM1 are shown. The sizes of the spherical nodes represent the enrichment factor that indicates the ratio of target genes of miRNAs enriched in the pathway among genes annotated in the pathway. The colors of the spherical nodes represent the classification of pathways based on the same participating genes

KEGG enrichment analysis revealed that a total of 121 pathways were potentially regulated by the miRNAs in subcluster H3-K17-SOM1 during *T. gondii* infection, and the main enriched KEGG pathways is shown in Fig. 5. The 28 KEGG pathways involved in human diseases, signal transduction, immune system, cellular processes, and metabolism were significantly enriched (*q* < 0.05) by the subcluster (Additional file 12: Table S11). Of these, three immune system-related KEGG pathways, including the chemokine signaling pathway (ssc04062), B cell receptor (BCR) signaling pathway (ssc04662), and C-type lectin receptor (CLR) signaling pathway (ssc04625), were not found to be significantly enriched by any subcluster of *K*-means clustering, SOM clustering, or hierarchical clustering.

Reactome enrichment analysis showed that 12 pathways were significantly enriched (*q* < 0.05) by the subcluster H3-K17-SOM1, of which five pathways were classed into immune system, especially cytokine signaling in immune system, following by three metabolism- and two cell cycle-related pathways (Additional file 12: Table S11).

**Fig. 6:**
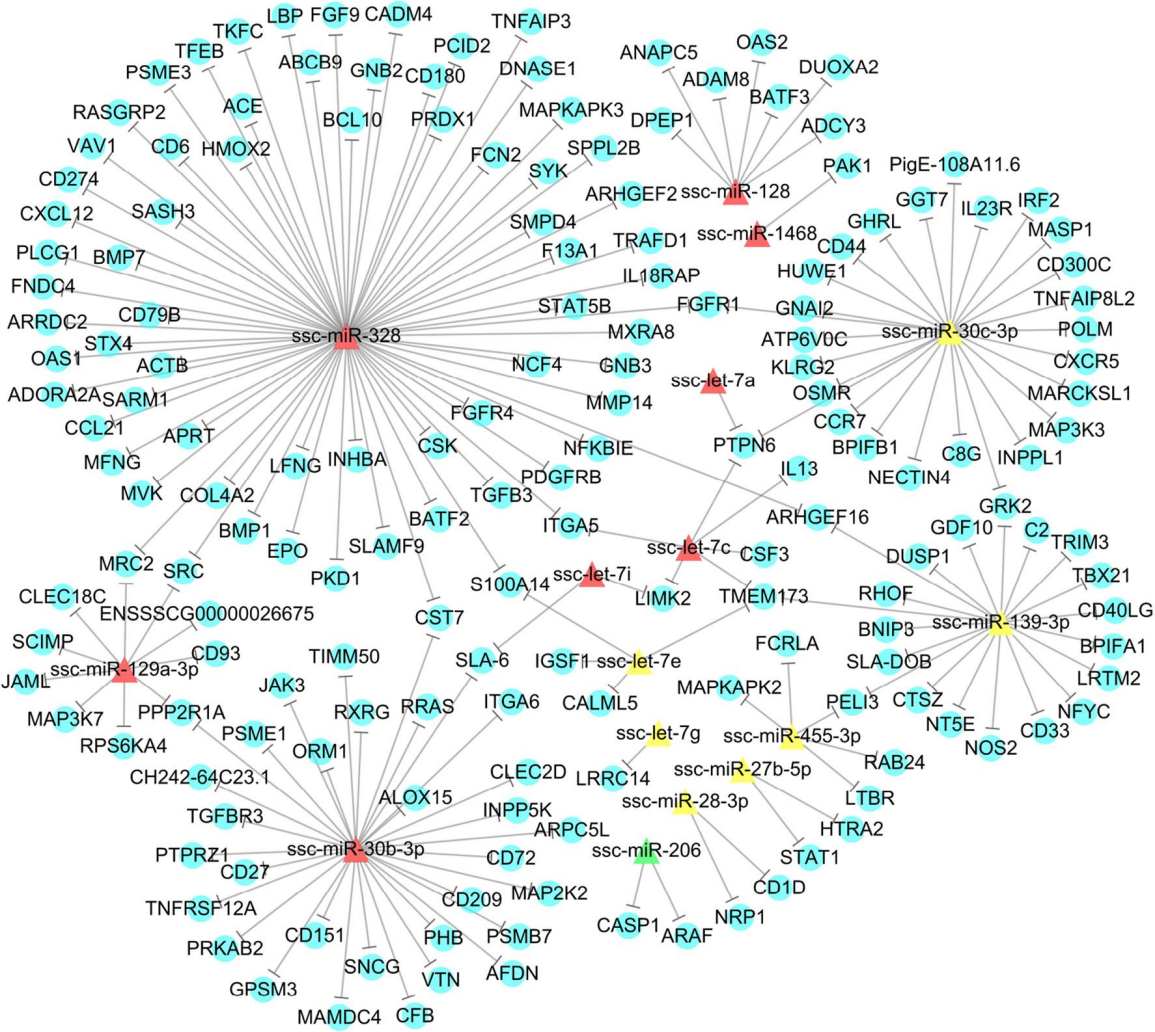
Regulatory network analysis of the interaction between the miRNAs in subcluster H3-K17-SOM1 and their potential immune-related target genes. The node shapes represent the miRNAs or target genes, which are connected by edges (negative interaction between miRNA to target gene). The colors of triangular nodes represent the significantly downregulated (red) and decreased (yellow) genes in the porcine spleen at 50 dpi, and significantly downregulated (green) genes in the porcine spleen at 10 dpi

A regulatory network was constructed for the immune-related target genes and miRNAs in subcluster H3-K17-SOM1 (Fig. 6). A total of 174 target genes encoding the different cytokines involved in immune regulation of the host, including chemokines, ILs, TNF, IFN, TLR, C-type lectin, and immunoglobulin, were potentially regulated by 16 miRNAs. Among these miRNAs, ssc-miR-328 regulated the greatest number of target genes, with 70 targets, following by ssc-miR-30b-3p regulating 31 target genes. The target genes regulated by the greatest number of miRNAs were *PTPN6* and *TMEM173*, each regulated by three miRNAs (Fig. 6). These results indicate that these miRNAs showing the downregulated expression at 50 dpi and cytokines might play central roles in the host response to *T. gondii* infection.

## Discussion

In the early phase of *T. gondii* infection, tachyzoites exist in almost all organs and tissues of pigs, which causes histopathological changes and triggers a broad host immune defense mechanism. During the course of infection, cytokine responses involving host immunity are altered by *T. gondii* to establish chronic infection. Studies have found that changes in the miRNA expression profiles of infected host cells contribute crucially to the interactions between the host and *T. gondii* [6, 9, 15–17]. In fact, this study is a continuing report based on our previous study on comparison of splenocyte microRNA expression profiles of pigs during acute and chronic toxoplasmosis [15]. In this study, many miRNA groups showing similar expression patterns at the different infection stages were obtained by the three clustering algorithms. The functional enrichment not only highlighted the gene clustering of BP such as metabolic regulation, ion transport, biosynthesis, and cell apoptosis induced by *T. gondii*, but also successfully revealed the key roles in the immunomodulatory mechanisms against *T. gondii* infection, especially those functional terms that are subcluster-specific.

### *K*-means clustering

In *K*-means clustering, compared with the other subclusters, subclusters K1, K2, K12, K14, and K15 displayed more specific terms involved in immune regulation after *T. gondii* infection (Additional file 2: Table S1). This study found that ssc-miR-124a in subcluster K1 regulated the IL-1-mediated signaling pathway by potentially targeting *TNIP2* and *RPS6KA4*. As a crucial modulator of immunity and inflammation [24], miR-124a has been reported to be abnormally expressed in multiple infectious inflammations [25]. The exogenous stimulation could induce miR-124a expression in most cases, which in turn functions as a negative regulator to control inflammation [26, 27]. A study showed that regulation of the inflammatory response by miR-124a was related to the expression of IL-1 [28], which may be one of the important immune regulatory mechanisms during chronic *T. gondii* infection to attenuate excessive inflammation through downregulation of IL-1 expression by ssc-miR-124a, with increased expression at 50 dpi. Also, the IL-1-mediated signaling pathway regulated by ssc-miR-124a may be associated with the inactivation of the Notch signaling pathway. Notch signaling has a central role in cell fate specification and differentiation [29], and has been demonstrated to be play an important role in various facets of *T. gondii* pathogenesis [30]. Previous studies reported that miR-124a was inversely associated with the inactivation of Notch signaling [31]. The introduction of miR-124a in neural progenitor cells significantly reduced *JAG1* transcript and protein levels, leading to inactivation of Notch signals [32]. Notch signaling was also found to negatively regulate the TLR-triggered inflammatory response, while inactivation of Notch signaling increased the secretion of inflammatory cytokines, including TNF-α, IFN-β, IL-6, and IL-1β [33]. *DLX2* transcription factor, which has been shown to have an important role in regulating Notch signaling, is a known target of miR-124 [34]. Therefore, the potentially interactive combinations of ssc-miR-124a/*DLX2* and even ssc-miR-4332/*BMP7* and *GATA2* in subcluster K1 in this study (Additional file 13: Table S12) may have key roles in negative regulation of the Notch signaling pathway in pig spleen tissue infected with *T. gondii*. These results seem to suggest a possible mechanism whereby chronic *T. gondii* infection downregulated the IL-1-mediated cellular response through Notch signaling regulated by the miRNAs and their targets in subcluster K1.

**Fig. 7:**
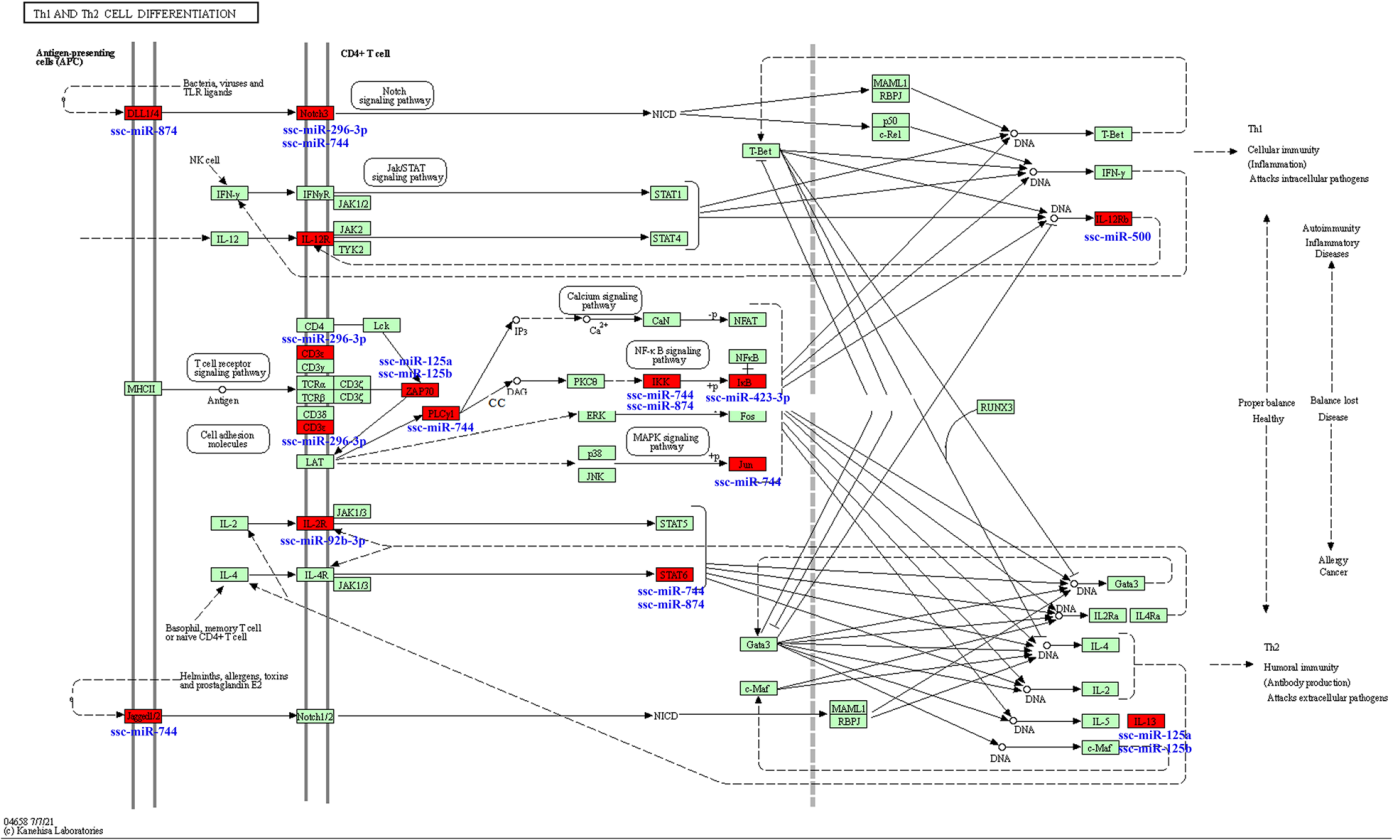
miRNAs in subcluster K2 involved in regulation of Th1 and Th2 cell differentiation pathways. The target genes marked by red nodes were found to be potentially regulated by the corresponding miRNAs

Furthermore, Th1 and Th2 cell differentiation (ssc04658) enriched only by the subcluster K2 in *K*-means clustering can be induced by Notch signaling [35, 36] through notch receptors Notch1/2 or Notch3 receiving signals from Jagged-like ligand Jagged1/2 and Delta-like ligand DLL4, respectively. Eight miRNAs in subcluster K2 were found to regulate Th1/Th2 cell differentiation by potentially targeting 14 genes of this pathway (Fig. 7). Of these, both *NOTCH3* (potentially targeted by ssc-miR-296-3p and ssc-miR-744) and its cell-bound ligand *DLL4* (potentially targeted by ssc-miR-874) were regulated by subcluster K2 to influence the polarization of Th1/Th2 responses during *T. gondii* infection. Studies on animal models of Th1/Th2 imbalance have demonstrated that Th1-polarized inflammation is associated with excessive destruction, while Th2 polarization is associated with excessive repair [37, 38]. Therefore, it is plausible that the decreased expression of miRNAs including ssc-miR-296-3p, ssc-miR-744, and ssc-miR-874 in subcluster K2 at 50 dpi may be an important regulatory factor in Th1/Th2 cell differentiation by potentially increasing DLL4/NOTCH3 signaling. In addition, our study found that *IL12RB1* might be targeted by the upregulated ssc-miR-500 at 10 dpi, and *IL13* potentially targeted by ssc-miR-125a and ssc-miR-125b, with a slight decrease in expression at 25 dpi (Fig. 7). This suggests that Th1 polarization of the host may have been inhibited to avoid excessive damage in acute infection, and then promoted the differentiation of Th2 cells and activated humoral immune responses based on antibody protection at 25 dpi. Previous studies showed that inflammatory injury of the host tissues caused by infection may be attributable to the dysregulation of the Th1 or Th2 immune response [39, 40]. Our analysis revealed an effective immune adjustment strategy of the host in the early stage of *T. gondii* infection, and miRNAs in subclusters K1 and K2 were involved in regulating the immune levels of the host resistance against persistent *T. gondii* infection through IL-1 cytokine responses and Th1/Th2 cell differentiation, partly dependent on Notch signaling transduction mechanisms.

According to clustering results, all the miRNAs of subcluster SOM17 were shared by subcluster K15, and both of the subclusters enriched into more immune-related GO terms compared with the other subclusters. Of note, a total of 16 target genes of innate immune response (GO: 0045087) were potentially regulated by seven miRNAs in subcluster K15, of which five targets including *TRAF3*, *TLR4*, *S100A14*, *IRF1*, and *TMEM173* were found to be involved in activation of innate immune response (GO: 0002218). Reactome pathway analysis further confirmed this finding, showing that the innate immune system (R-SSC-168249) was also significantly enriched by subcluster K15 (Additional file 14: Table S13). Twenty-five target genes in this pathway were potentially regulated by seven miRNAs of subcluster K15. Among the 14 branch pathways of the innate immune system, 12 pathways were found to be regulated, of which the most important branch pathway was the neutrophil degranulation pathway (R-SSC-6798695), with 14 genes potentially regulated by six miRNAs (ssc-miR-4334-3p, ssc-miR-339-3p, ssc-miR-17-3p, ssc-miR-9851-3p, ssc-miR-15a, and ssc-miR-1285), followed by TLR cascade (R-SSC-168898) with seven genes potentially regulated by five miRNAs (ssc-miR-4334-3p, ssc-miR-339-3p, ssc-miR-9851–3, pssc-miR-17-3p, and ssc-miR-17-5p). Neutrophils are primary effector cells of innate immunity, rapidly recruited to defend the host against invading pathogens. In response to infection, neutrophils leave the circulation, migrate toward the inflammatory focus, and fight infection by phagocytosis and degranulation [41]. Neutrophil-depleted mice infected with *T. gondii* succumb to death during the acute phase of infection [42], indicating that the neutrophil-mediated innate immune response plays a key role in the outcome of mice following *T. gondii* infection. Of seven genes in the TLR cascade pathway, five including *PTPN4*, *TRAF3*, *S100A12*, *RIPK1*, and *TLR4* were enriched into the TLR4 cascade pathway (R-SSC-166016), and were mainly involved in MyD88-independent TLR4 cascade (R-SSC-166166) under potential regulation of ssc-miR-339-3p, ssc-miR-4334-3p, and ssc-miR-9851-3p. A previous study showed that expression of TLR4 and MyD88 was markedly increased in *T. gondii*-infected mice tissues [43]. Our results also support a previous finding that TLR4/MyD88 signaling can be activated by *T. gondii* [44], and the TLR4/MyD88 signal cascade stimulates distinct pathways leading to the production of TNF-α and pro-inflammatory cytokines to amplify the inflammatory activity of the host [43, 44], suggesting that ssc-miR-339-3p, ssc-miR-4334-3p, and ssc-miR-9851-3p are potentially central regulators of the inflammatory response of the host during *T. gondii* infection. These analyses indicate that *T. gondii* induced a potent innate immune response mainly involving neutrophil degranulation and TLR4 cascade signaling through the differential expression of miRNAs in subcluster K15.

### SOM clustering

Compared with *K*-means clustering, SOM clustering revealed distinct cellular processes and signaling pathways related to immune responses regulated by miRNAs during different stages of *T. gondii* infection. The GO terms involving B cell activation were enriched by the miRNAs in subclusters SOM17, SOM1, and SOM25. However, the regulatory mechanisms on B cell activation of these three subclusters are different due to the distinct regulators. Three, three, and ten miRNAs in subclusters SOM17, SOM25, and SOM1 were found to potentially regulate eight, seven, and 14 target genes that relate to B cell activation, respectively (Additional file 15: Table S14). Of these genes, matrix metalloproteinase-14 (*MMP14*), involved in B cell differentiation and maintaining normal B cell development [45], is the only gene shared among the three subclusters. The B cell activation-related-miRNAs of subcluster SOM17 (ssc-miR-4334-3p, ssc-miR-9851-3p, and ssc-miR-339-3p) were mainly downregulated in expression at 10 dpi, while SOM1 (ssc-miR-328, novel-232, ssc-miR-504, ssc-let-7c, ssc-miR-30c-3p, ssc-miR-671-3p, ssc-let-7a, ssc-miR-139-3p, ssc-miR-129a-3p, and ssc-miR-125a) and SOM25 (ssc-miR-127, ssc-miR-370, and novel-262) were mainly downregulated in expression at 50 dpi. Also, the miRNAs in subcluster SOM25 showed a slightly upregulated expression at 10 dpi. *MMP14*, *CD40*, and *IL13* in positive regulation of B cell activation (GO: 0050871) were potentially targeted by SOM17, while *MMP14*, *TAT5B*, *TGFB1*, and *GPR183* in positive regulation of B cell activation (GO: 0050871) were potentially targeted by SOM25 (Additional file 15: Table S14). Therefore, the potential target genes regulated by subcluster SOM17 were distinct to subcluster SOM25, which might represent different regulatory pathways related to B cell activation induced by acute infection. It is still unclear whether the regulatory trends in B cell activation were induced by miRNAs during acute infection due to the opposite expression patterns between subclusters SOM17 and SOM25 at 10 dpi. CD8^+^ T cells with IFN-γ production have been demonstrated to be the main effector cells and play the most significant role in protection against acute *T. gondii* infection [46, 47]. Nonetheless, this study still found that the B cell activation signaling pathway was facilitated under the regulation of a few miRNAs and their target genes, indicating a possible role of B cells in acute *T. gondii* infection. Furthermore, chronic infection may result in the positive regulation of B cell activation through the downregulated expression of more miRNAs at 50 dpi in SOM1 and SOM25 (Additional file 15: Table S14). In resistance to persistent active infection with *T. gondii*, the two subclusters induced a functional aggregation of target genes to regulate B cell activation and proliferation, suggesting that B cells play an important role during chronic *T. gondii* infection, most likely through their production of specific antibodies [48].

In addition, leukocyte migration and chemokine activity were also found to be induced by *T. gondii* in this study. A series of studies have reported that chemokines exert critical roles in various diseases including toxoplasmosis [49]. Compared to the other subclusters of SOM clustering, our study found that leukocyte migration (GO: 0050900) and chemokine activity (GO: 0008009) were specifically enriched by SOM9. *CDC42*, *CXCL12*, *VEGFD*, *CCL25*, *CXCL14*, and *CXCL16* involved in leukocyte migration and chemokine activity were potentially regulated by ssc-miR-185, ssc-miR-29a, and ssc-miR-103 in subcluster SOM9 (Additional file 16: Table S15). A previous study showed that acute toxoplasmosis is associated with higher levels of several circulating cytokine/chemokine mediators, including CXCL12 and CCL25 [50]. CCL25 has been widely recognized as mucosal chemokines and recruits certain important subsets of T cells that express CCR9 to the small intestine [51]. CXCL12 binding CXCR4 is known to mediate the recruitment of T lymphocytes and enhance their stimulation through the T cell receptor [52]. However, CXCL14 can also bind CXCR4, inhibiting the migration of cells in response to the CXCL12–CXCR4 axis [53]. Thus, the proposed function for CXCL14 is immune surveillance, as it is able to act as chemoattractant to macrophages, immature dendritic cells, and mast cells [51], and might have the potential to regulate the function of the CXCL12–CXCR4 signaling axis or play a compensatory role when CXCL12 is absent [54]. It is very obvious that the chemokine system has a critical role in the response to the initial stages of *T. gondii* infection, allowing establishment of an effective innate immune response to the parasite [49]. In this study, *CXCL12*, *CCL25* and *CXCL14* were potentially regulated by ssc-miR-185 with a significant upregulated expression at 10 dpi, suggesting a possible mechanism of the host to maintain homeostasis and avoid hyper-immune reactions in tissues highly exposed to *T. gondii*.

### Hierarchical clustering

According to this study, compared to *K*-means clustering and SOM clustering, each subcluster for hierarchical clustering showed more miRNA members, and as a result, the functional aggregation of target genes of miRNAs was relatively insignificant. Nonetheless, H-subcluster-specific KEGG pathways related to immune responses against *T. gondii* infection were still found, especially in subcluster H2. As previously reported [30, 55, 56], cytokine–cytokine receptor interaction (ssc04060) was found to be involved in immunomodulation of the host during acute and chronic *T. gondii* infection in the current study. A total of 52 genes of the pathway involving chemokines including CC and CXC subfamilies, class I helical cytokines, class II helical IL10/28-like cytokines, IL1-like cytokines, IL17-like cytokines, TNF family, and TGF-β family were potentially targeted by 31 miRNAs in subcluster H2 (Additional file 17: Table S16). Interestingly, more interactions in chemokines, class I helical cytokines, and TNF family were found to be induced by *T. gondii* infection. Also, the majority of the enriched chemokine-related genes encoded ligand protein, while the enriched genes related to class I helical cytokines and the TNF family mainly encoded receptor protein. These findings suggest that *T. gondii* infection influenced a large number of cytokine-mediated signaling transduction processes of the host, especially the cellular process and pathways induced by class I helical cytokines and the TNF family, which have been shown to play important roles in regulating key immune processes including host defense, lymphoid organogenesis, acute immune response, and inflammation [57, 58]. Of note, this study found that *IL17F*/*IL17RC* were potentially commonly regulated by the miRNAs, with increased expression at 10 dpi (ssc-miR-7134-3p, ssc-miR-500, and ssc-miR-185) and decreased expression at 50 dpi (ssc-miR-432-3p, ssc-miR-1343, and ssc-miR-1307). Additionally, *IL10RA* and *IL20RB* were potentially regulated by the miRNAs, with decreased expression at 50 dpi (ssc-miR-296-3p, ssc-miR-2366, and ssc-miR-1343) (Additional file 17: Table S16). IL17F exerts a crucial function in host defense against infections [59], and IL17 receptors are present on many cell types including immune cells. Their stimulation induces the expression of various cytokines and chemokines, leading most prominently to recruitment and maturation of neutrophils [60]. Additionally, IL-10 is a critical cytokine used by immune cells to suppress inflammation. A previous study showed that ocular toxoplasmosis induces upregulation of IL-10 to prevent unnecessary tissue damage, and moderate upregulation of IL-17 to control parasite proliferation [61], showing that IL-10 combined with IL-17 plays a role in regulating the immune status of the host during *T. gondii* infection. At the same time, of the 31 miRNAs potentially regulating the cytokine–cytokine receptor interaction pathway in subcluster H2, almost all miRNAs showed increased expression at 10 dpi or decreased expression at 50 dpi. This indicates that the interactions amongst cytokines play a crucial role in the balance of the immune microenvironment during the different infection stages, such as preventing excessive immunity in acute infection and maintaining the level of inflammation to control the reactivation of *T. gondii* in chronic infection. In addition, several previous researchers found that the cytokine–cytokine receptor interaction pathway was also influenced by different *T. gondii* strains during challenge in various infection models including cat, mouse, and human cells [30, 55, 56], suggesting a common feature wherein interactions between cytokines and cytokine receptors have extremely important roles in the host immune responses against *T. gondii*, and are not strictly host-specific or strain-specific. Nonetheless, our analysis revealed that miRNAs in subcluster H2 involved in the cytokine–cytokine receptor interaction pathway may be the important immunomodulatory molecules of the host during *T. gondii* infection.

### Subcluster H3-K17-SOM1

Subclusters obtained by the three algorithms were compared to identify miRNAs with similar expression patterns. This study found that 22 miRNAs were shared by subcluster K17 of *K*-means clustering, subcluster SOM1 of SOM clustering, and subcluster H3 of hierarchical clustering, which confirmed our clustering outputs to be useful and reliable. Although the target genes of miRNAs in subcluster H3-K17-SOM1 were enriched to many terms of cellular components and molecular functions, such as mitochondrion, zinc ion transmembrane transporter activity, and protein binding, a higher proportion of target genes were found to be annotated to BP, and reflected a trend toward clustering of immune processes. A large number of target genes were involved in the regulation of inflammatory response, TNF production, antigen processing, and presentation and complement activation, particularly T cell and B cell differentiation and proliferation, IL production, and immune cell migration and chemotaxis. Previous research has shown that the production of pro-, anti-, and pro-/anti-inflammatory ILs are activated and have a key role in resistance to *T. gondii* in different infection stages [62], which is consistent with our finding. This study found that *T. gondii* induced and regulated the production of pro-inflammatory ILs, including IL-1, IL-2, IL-8 and IL-12, anti-inflammatory ILs including IL-4 and IL-10, and pro-/anti-inflammatory IL-6 by potentially altering expression of miRNAs (ssc-miR-328, ssc-miR-30c-3p, ssc-miR-129a-3p, ssc-miR-139-3p, ssc-miR-30b-3p, ssc-let-7a, ssc-let-7c) in subcluster H3-K17-SOM1 (Additional file 18: Table S17). In particular, the function of IL-6, as a Th2 type cytokine, is extremely complex. On one hand, IL-6 can promote proliferation and differentiation of T cells and B cell differentiation into immunoglobulin-producing cells [63, 64], and facilitate the production and regulation of IL-17 from NK cells during acute toxoplasmosis [65]; on the other hand, it enhances intracellular replication of *T. gondii* and reverses IFN-γ-mediated activation of murine peritoneal macrophages [66]. This study found that up to nine target genes corresponding to seven miRNAs were involved in the regulation of IL-6 production, showing that a role for IL-6 in the context of *T. gondii* infection may be of particular importance. IL-12, produced by various types of cells including monocytes, neutrophils, macrophages, conventional dendritic cells (cDCs), and plasmacytoid DCs [67], is a pivotal cytokine to mediate inflammation and control parasites during toxoplasmosis by enhancing T and NK cell cytotoxicity and inducing IFN-γ production to drive a Th1 type response [68, 69]. Additionally, IL-2, as a classical Th1 type cytokine, plays a dominant role in promoting the expansion and survival of Treg cells [70], which play a major role in the preservation of host tissue integrity during *T. gondii* infection [71]. A recent study showed that adenovirus co-expressing CD40 ligand and IL-2 can stimulate the maturation of DCs and high levels of IL-12 production [72]. However, IL-12 production can be inhibited by enhancing the production of Th2 type cytokines such as IL-4 and IL-10 [62]. In this study, ssc-miR-139-3p/*CD40LG* was found to be commonly involved in positive regulation of IL-12, IL-4, and IL-10 production (Additional file 18: Table S17), indicating a possible shared regulatory mechanism for pro- or anti-inflammatory response to maintain adaptive immunity of the host during chronic *T. gondii* infection. At the same time, our results support the idea that immune suppression following activation is also an important step to prevent immunopathology during *T. gondii* infection [62]. Reactome pathway analysis further confirmed the regulatory effect of miRNAs in subcluster H3-K17-SOM1 on the ILs and their receptor signaling pathways during *T. gondii* infection. Signaling by the IL pathway (R-SSC-449147) was found to be significantly enriched by the subcluster. Of the 11 genes regulated by the IL pathway, seven (*ACTB*, *ARAF*, *CSF3*, *CSK*, *FGF9*, *MAP2K2*, and *PTPRZ1*) were found to be involved in other IL signaling (R-SSC-449836), and the other genes were involved in IL-1 signaling (R-SSC-9020702), IL-17 signaling (R-SSC-448424), IL-2 family signaling (R-SSC-451927), IL-3, IL-5, and GM-CSF signaling (R-SSC-512988), IL-7 signaling (R-SSC-1266695), and IL receptor SHC signaling (R-SSC-912526). Of note, ssc-miR-30b-3p/*JAK3* was found to potentially regulate the last three pathways listed above. *JAK3* is involved in the signaling pathways of various cytokines (e.g., IL-2, IL-4, IL-7, IL-9, IL-15, and IL-21) which play crucial roles in T cell differentiation, proliferation, and survival [73]. Thus, this may be an important mechanism for *T. gondii* to coordinate various cytokine responses of the host during the infection. In particular, all the miRNAs, including ssc-miR-30c-3p, ssc-miR-328, ssc-miR-30b-3p, ssc-miR-129a-3p, and ssc-let-7c, involved in IL pathway signaling were also found to potentially regulate IL production, suggesting their pivotal regulatory roles in IL-mediated signaling pathways triggered by *T. gondii*.

Inflammatory responses can be mediated through ILs produced by immune cells, which control migration and residence to cause local accumulation by chemokines and their receptors. As is known, CC chemokines mainly stimulate monocytes, but also basophils, eosinophils, T lymphocytes, and NK cells. The other family, CXC, mainly stimulates neutrophil chemotaxis [74]. In the current study, chemotaxis of immune cells including neutrophils, lymphocytes, dendritic cells, endothelial cells, eosinophils, monocytes, and leukocytes was found to be potentially regulated by the miRNAs (ssc-miR-328, ssc-miR-128, ssc-miR-129a-3p, ssc-let-7e, ssc-miR-28-3p, and ssc-miR-30c-3p) in subcluster H3-K17-SOM1. Notably, ssc-miR-328/*CCL21* was potentially involved in the chemotaxis of neutrophils, lymphocytes, dendritic cells, eosinophils, and monocytes, which are important cells that prevent *T. gondii* infection [75], and ssc-miR-30c-3p was also found to regulate chemokine receptor-induced signaling pathways through potential targeting of *CCR7* and *CXCR5*. A significant increase in the expression of CCL19, CCL21, and CCR7 has been observed in peripheral and central nervous system (CNS) tissues over the course of *T. gondii* infection [76]. CCL21 exhibits a high affinity for CCR7, which is expressed on T cells and mature dendritic cells [77], and its expression contributes to T cell-mediated CNS pathology by facilitating CD4^+^ T cell migration into parenchymal sites following pathogenic insults to the CNS following *T. gondii* infection [78]. CCR7 was found to be an absolute requirement for protective immunity to *T. gondii* by enabling T cell priming and increasing IFN-γ production, while *CCR7*(−/−) mice succumbed to the parasite early in the acute phase of infection [76]. Unlike CCL21/CCR7, which stimulate T cell priming and activation and lymphocyte recruitment to inflamed tissue [74], CXCR5 and its ligand CXCL13 have been associated with potent B-cell-activation capability for humoral immune response [79]. It has been shown that the expression levels of CXCR5 were very low during murine ocular toxoplasmosis [80]. Nevertheless, toxoplasmic encephalitis led to a high level of expression of CXCR5, which induced a strong and sustained antibody-mediated immunity through involvement in antibody production [81]. This evidence indicates that miRNAs in subcluster H3-K17-SOM1 not only coordinated the migration and recruitment of inflammatory cells, but was involved in cellular and humoral immune processes after *T. gondii* infection by regulating the expression of chemokines and their receptors. The significant enrichment for the chemokine signaling pathway (ssc04062) by subcluster H3-K17-SOM1 also supports and corroborates this view. A total of 16 genes in the chemokine signaling pathway were potentially regulated by eight miRNAs in subcluster H3-K17-SOM1 to influence a series of cellular processes, including leukocyte transendothelial migration, chemotaxis, cytokine production, and cellular growth and differentiation, in response to chronic *T. gondii* infection. In addition to this pathway, the other two immune-related KEGG pathways [BCR signaling pathway (ssc04662) and C-type lectin receptor signaling pathway (ssc04625)] were also significantly enriched by this subcluster.

**Fig. 8:**
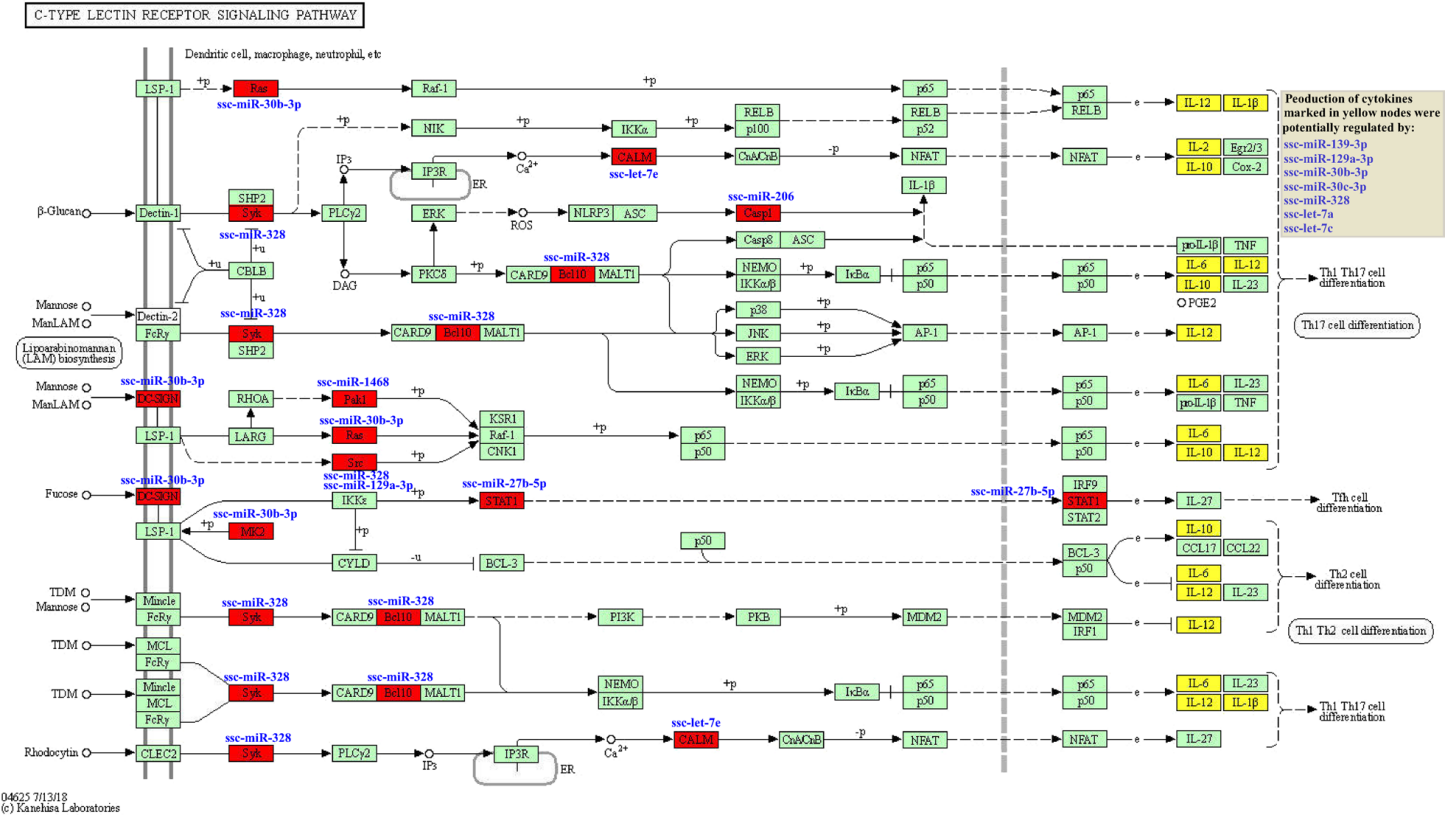
miRNAs in subcluster H3-K17-SOM1 involved in the regulation of the C-type lectin receptor signaling pathway. The target genes marked by red nodes were found to be potentially regulated by the corresponding miRNAs

The C-type lectin receptor (CLR) signaling pathway is associated with Th cell differentiation by influencing two important immune-related pathways, namely Th1 and Th2 cell differentiation and Th17 cell differentiation, which were enriched by subcluster K2 and subcluster SOM25, respectively. Upon ligand binding, CLRs stimulate intracellular signaling cascades that induce the production of inflammatory cytokines and chemokines [82], including IL-1, IL-2, IL-12, IL-10, and IL-6 [82, 83]. These coincided with the GO enrichment result of the subcluster H3-K17-SOM1, indicating that the function of miRNAs in this subcluster may focus on regulating cytokine production through the CLR signaling pathway, and further regulating Th cell differentiation in cooperation with subcluster K2 and subcluster SOM25, consequently triggering innate and adaptive immunity to *T. gondii*. Of these miRNAs, ssc-miR-328, ssc-miR-30b-3p, and ssc-miR-129a-3p, which are potentially involved in regulating the CLR signaling pathway and IL production, may undoubtedly play a prominent role (Fig. 8). Notably, *SYK*/*BCL10* is the central axis of the pathway and can induce NF-κB activation [84], showing that the potential regulation axis ssc-miR-328/*SYK*/*BCL10* may play a crucial role via regulation of NF-κB signaling in CLR-mediated immunity against chronic *T. gondii* infection. Moreover, their interaction may have an important role in the BCR signaling pathway to regulate humoral immunity during *T. gondii* infection. Additionally, a previous study found that DC-SIGN (also known as CD209) plays an active role in *T. gondii* invasion and host dissemination [85]. The present study revealed that ssc-miR-30b-3p is a potential regulator of *DC-SIGN* (Fig. 8), which shows that the downregulation of this miRNA may be beneficial to the reactivation of *T. gondii* during chronic infection.

In addition to the abovementioned regulators and their targets in subcluster H3-K17-SOM1, other interactions between miRNAs and immune-related target genes were revealed by a regulatory network in this study (Fig. 6). The most striking target genes, *PTPN6* and *TMEM173*, might be the important nodes involved in the host response to *T. gondii*. Src homology region 2 domain-containing phosphatase 1 (SHP-1), encoded by the *PTPN6* gene, is a key negative regulator of immune cell function [86, 87]. GO and KEGG analysis showed that *PTPN6* was involved in negative regulation of various immune processes, including TNF production, IL-6 production, T cell proliferation, and T cell receptor (TCR) and BCR signaling pathways. *PTPN6* is also known to negatively regulate IL-1R/TLR-mediated production of proinflammatory cytokines by suppressing the activation of mitogen-activated protein kinases (MAPKs) and the transcription factor NF-κB [88], similar to the regulatory mechanism of *PTPN6* on B cell immunity through the BCR signaling pathway during *T. gondii* infection. At the same time, *PTPN6* negatively regulates the tyrosine phosphorylation of SYK [89], which may play a crucial role in the CLR and BCR signaling pathways to regulate cellular and humoral immunity in chronic *T. gondii* infection. A previous study found that SHP-1 expression was upregulated in *T. gondii*-infected NK cells to inhibit cytotoxicity and cytokine production [90], which is consistent with the expression trend of *PTPN6* potentially controlled by ssc-let-7a and ssc-let-7c, showing a downregulated expression trend at 50 dpi in the current study and suggesting their negative regulatory role in immune response during chronic *T. gondii* infection. Stimulator of interferon genes (STING, encoded by *TMEM173*) is an endoplasmic reticulum (ER)-associated immune adaptor protein that facilitates innate immune signaling [91]. *TMEM173* is critical in initiating type I IFN and amplifying the inflammatory response during infection and tissue injury [92]. A recent study showed that mice deficient in STING (Sting^gt/gt^ mice) are much more susceptible to *T. gondii* infection than wild-type mice. Of note, the induction of inflammatory cytokines, type I IFN, and IFN-stimulated genes in the spleen of Sting^gt/gt^ mice was significantly impaired [93]. This study demonstrated that ssc-let-7c and ssc-let-7e showed a downregulated expression trend at 50 dpi potentially targeting the *TMEM173* gene, indicating their roles for the regulation of type I IFN-dependent innate immunity and inflammatory responses during chronic *T. gondii* infection. miRNAs of the let-7 family can trigger innate immune response to enhance the host defense ability [94]. These analyses revealed a prominent role of the let-7 family in regulating innate immunity induced by chronic *T. gondii* infection. Altogether, control of *T. gondii* infection may involve the coordinated action of both cellular and humoral immunity through the miRNAs in subcluster H3-K17-SOM1 to ensure stage conversion and establishment of chronic infection [81].

## Conclusions

In summary, we observed dynamic changes in the expression of miRNAs in pig spleen tissues from acute to chronic infection, and obtained multiple miRNA groups with similar expression patterns at different infection stages through three clustering algorithms. Functional analysis of target genes was performed by GO, KEGG, and Reactome pathway analysis. Here, we focus on the function of the miRNA groups in immune regulation. A large group of immunomodulatory signaling molecules were potentially controlled by these miRNA groups to regulate multiple immune processes, for instance, IL-1-mediated cellular response and Th1/Th2 cell differentiation partly depending on Notch signaling transduction for subclusters K1 and K2, innate immune response involved in neutrophil degranulation and TLR4 cascade signaling for subcluster K15, B cell activation for subclusters SOM17, SOM1, and SOM25, leukocyte migration and chemokine activity for subcluster SOM9, cytokine–cytokine receptor interaction for subcluster H2, IL production, chemotaxis of immune cells, the chemokine signaling pathway, and the CLR signaling pathway for subcluster H3-K17-SOM1. These may reflect the key regulatory properties of subcluster miRNA molecules and the important features in the immune regulation induced by acute and chronic infections of *T. gondii*. These results not only contribute to our understanding of the host immunomodulatory mechanisms induced by *T. gondii* infection, but also offer new insight into the identification of physiological immune responses and maintenance of tolerance in pig spleen tissues.

## Supplementary Information

**Additional file 1: Figure S1.** The PCA plot of individual small RNA libraries.

**Additional file 2: Table S1.** The significantly enriched GO terms for predicted target genes of miRNAs in different subclusters by *K*-means clustering.

**Additional file 3: Table S2.** The significantly enriched KEGG pathways for predicted target genes of miRNAs in different subclusters by *K*-means clustering.

**Additional file 4: Table S3.** The significantly enriched Reactome pathways for predicted target genes of miRNAs in different subclusters by *K*-means clustering.

**Additional file 5: Table S4.** The significantly enriched GO terms for predicted target genes of miRNAs in different subclusters by SOM clustering.

**Additional file 6: Table S5.** The significantly enriched KEGG pathways for predicted target genes of miRNAs in different subclusters by SOM clustering.

**Additional file 7: Table S6.** The significantly enriched Reactome pathways for predicted target genes of miRNAs in different subclusters by SOM clustering.

**Additional file 8: Table S7.** The significantly enriched GO terms for predicted target genes of miRNAs in different subclusters by hierarchical clustering.

**Additional file 9: Table S8.** The significantly enriched KEGG pathways for predicted target genes of miRNAs in different subclusters by hierarchical clustering.

**Additional file 10: Table S9.** The significantly enriched Reactome pathways for predicted target genes of miRNAs in different subclusters by hierarchical clustering.

**Additional file 11: Table S10.** The significantly enriched GO terms for predicted target genes of miRNAs in subcluster H3-K17-SOM1.

**Additional file 12: Table S11.** The significantly enriched KEGG and Reactome pathways for the predicted target genes of miRNAs in subcluster H3-K17-SOM1.

**Additional file 13: Table S12.** miRNAs in subcluster K1 potentially regulating the gene expression in the Notch signaling pathway (GO:0007219).

**Additional file 14: Table S13.** miRNAs in subcluster K15 regulating the gene expression in the innate immune system pathway (R-SSC-168249) enriched by the Reactome pathway.

**Additional file 15: Table S14.** miRNAs in subclusters SOM1, SOM17, and SOM25 regulating the gene expression in B cell activation (GO:0042113).

**Additional file 16: Table S15.** miRNAs in subcluster SOM9 regulating the gene expression in leukocyte migration (GO:0050900) and chemokine activity (GO:0008009).

**Additional file 17: Table S16.** miRNAs in subcluster H2 involved in regulation of the cytokine–cytokine receptor interaction pathway (ssc04060).

**Additional file 18: Table S17.** miRNAs in subcluster H3-K17-SOM1 regulating the expression of genes related to interleukin production.

## Data Availability

The data sets supporting the findings of this article are included within the article. Full details of the sequence data were submitted to the GEO public database (http://www.ncbi.nlm.nih.gov/geo/) with the GEO accession number GSE113130; the raw data are available in the NCBI Sequence Read Archive under the accession number SRP139950.

## Abbreviations

NF-κB: Nuclear factor kappa B
SOM: Self-organizing map
GO: Gene Ontology
KEGG: Kyoto Encyclopedia of Genes and Genomes
GEO: Gene Expression Omnibus
dpi: Days post-infection
TPM: Transcripts per million clean tags
DEMs: Differentially expressed miRNAs
BP: Biological processes
CC: Cellular components
MF: Molecular functions
TLR4: Toll-like receptor 4
CLR: C-type lectin receptor
BCR: B cell receptor
ILs: Interleukins
TNF: Tumor necrosis factor
IFN: Interferons
