# Supplementary figures and images for: Cluster analysis of splenocyte microRNAs in the pig reveals key signal regulators of immunomodulation in the host during acute and chronic Toxoplasma gondii infection
Source: Parasit Vectors. 2022 Feb 17;15:58. doi: 10.1186/s13071-022-05164-3 (PMC8851844; doi:10.1186/s13071-022-05164-3)

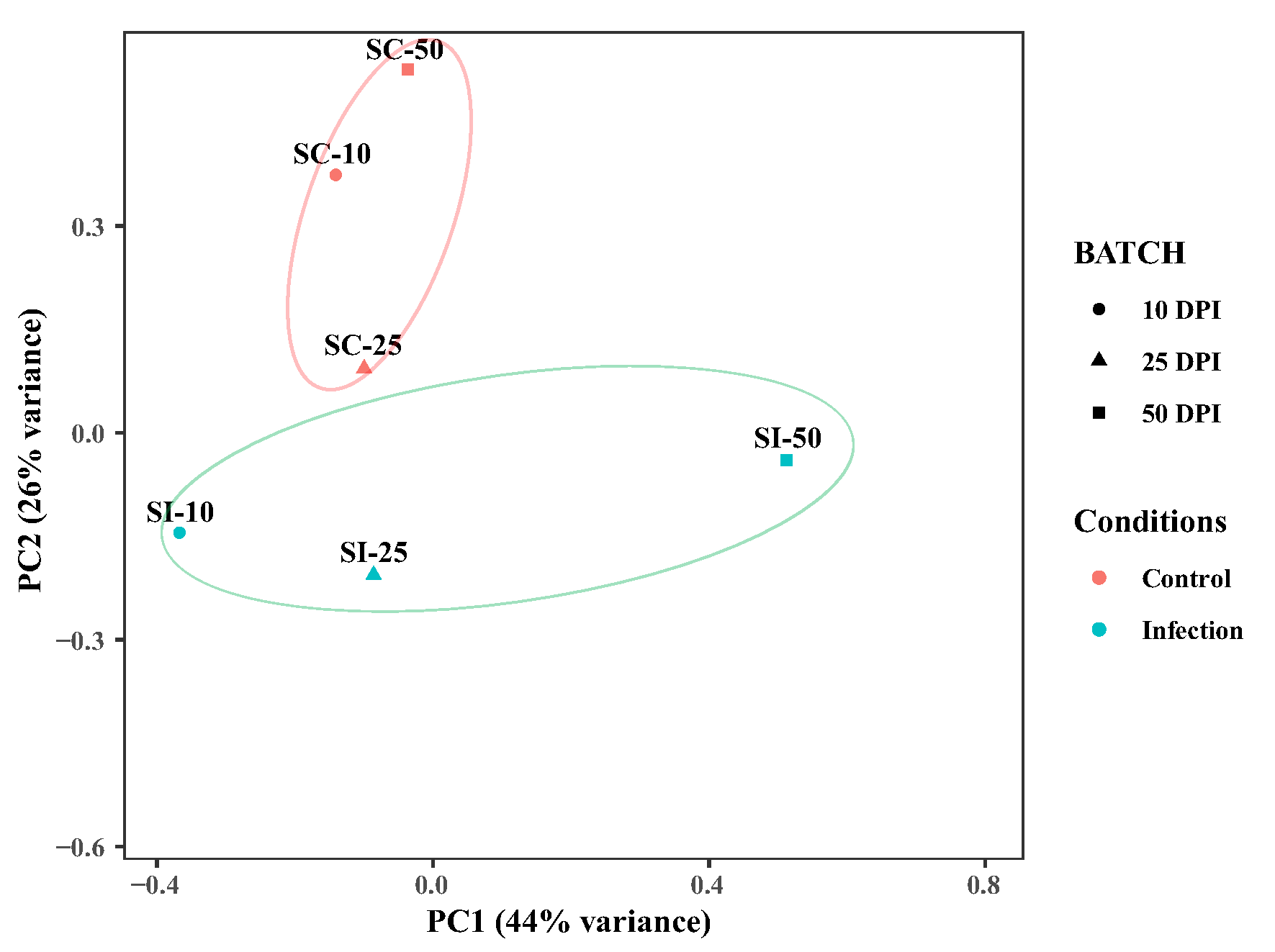

Supplement: Supplementary file 1 — Additional file 1: Figure S1. The PCA plot of individual small RNA libraries. [file 13071_2022_5164_MOESM1_ESM.tiff]
